# Emergency Department Experience with Novel Electronic Medical Record Order for Referral to Food Resources

**DOI:** 10.5811/westjem.2017.12.35211

**Published:** 2018-02-26

**Authors:** Marc L. Martel, Lauren R. Klein, Kurt A. Hager, Diana B. Cutts

**Affiliations:** * Hennepin County Medical Center, Department of Emergency Medicine, Minneapolis, Minnesota; † Tufts University School of Medicine and Friedman School of Nutrition Science and Policy, Boston, Massachusetts; ‡ Hennepin County Medical Center, Department of Pediatrics, Minneapolis, Minnesota

## Abstract

**Introduction:**

Food insecurity is a significant issue in the United States and is prevalent in emergency department (ED) patients. The purpose of this study was to report the novel use of an integrated electronic medical record (EMR) order for food resources, and to describe our initial institutional referral patterns after focused education and implementation of the order.

**Methods:**

This was a retrospective, observational study, describing food-bank referral patterns before and after the implementation of dedicated ED education on the novel EMR order for food resources.

**Results:**

In 2015, prior to formal education a total of 1,003 referrals were made to the regional food bank, Second Harvest Heartland. Five referrals were made from the ED. In 2016, after the educational interventions regarding the referral, there were 1,519 referrals hospital-wide, and 55 referrals were made from the ED. Of the 1,519 referrals 1,129 (74%) were successfully contacted by Second Harvest Heartland, and 954 (63%) accepted and received assistance.

**Conclusion:**

Use of the EMR as a tool to refer patients to partner organizations for food resources is plausible and may result in an increase in ED referrals for food resources. Appropriate education is crucial for application of this novel ED process.

## INTRODUCTION

The United States Department of Agriculture (USDA) defines food insecurity as “limited or uncertain availability of nutritionally adequate and safe foods, or limited or uncertain ability to acquire acceptable foods in socially acceptable ways”. 1 Food insecurity is prevalent in the U.S., affecting 12.7% of the population in 2016. 2 The impact of food insecurity can be pervasive and has been described by the World Health Organization as a key social determinant of health. 3

The prevalence of hunger and the impact of food insecurity in the emergency department (ED) is clinically important and likely underestimated. 4–7 One study reported that 23.7% of ED patients reported hunger or food insecurity in the year prior, and nearly 18% of patients chose medicine over food during that same time period. Many of these patients reported the belief that this decision resulted in illness, ED visits, and hospitalization. 7

To address such issues, healthcare institutions reportedly are partnering with food resources for their patients. 8 But if these services are to reach the patients, referrals must be initiated by staff or accessed directly by the patient/client. While our institution has partnered with the food bank Second Harvest Heartland since 2010, referrals from the ED were made infrequently. In 2015 a novel referral model was developed in the form of an electronic medical record (EMR) order by which ED providers could easily refer patients for food resources. Targeted education of the ED staff on food insecurity and instruction on the use of the EMR order was also performed.

The purpose of this study was to report the novel use of an integrated EMR order for food resources, and to describe our initial institutional referral patterns after focused education and implementation of the order.

## METHODS

### Study Setting and Population

This is a retrospective, observational study, describing food-bank referral patterns before (2015) and after (2016) the implementation of dedicated ED education on the novel EMR order for food resources. The study population was an urban, county ED with greater than 110,000 annual visits. This study was deemed exempt from review by our institutional review board.

### Description of Food Services and Food Bank

Feeding America is a network of food banks, food pantries, and meal programs providing food and services in the U.S. It is the nation’s largest organization to support hunger relief. Second Harvest Heartland, a midwestern member of Feeding America, is one of the nation’s largest food banks, and supports over 1,000 food shelves and other partner programs that distribute food to over 532,000 individuals in Minnesota and western Wisconsin annually.

Our institution and Second Harvest Heartland have partnered to provide food assistance to our patients since 2010. As a result of this relationship, healthy food is available in our clinics in the form of bagged groceries, and on site through an institutional food shelf. Patients are able to receive food through clinics, at discharge from an inpatient stay, or delivered to their homes through a community paramedic program or visiting nurses.

### The Institutional Referral Program

To determine eligibility for food services referrals in our institution, staff or providers screen patients for food insecurity with two questions. This two-question screening tool is a validated, abbreviated version of the 18-item U.S. Household Food Security Scale (HFSS), which is used to monitor national food security. 9 For purposes of our referral program, we modified the screening questions to be dichotomous yes/no responses from the validated responses: often, sometimes, or never. The questions included (1) “Within the past 12 months we worried whether our food would run out before we got money to buy more;” and (2) “Within the past 12 months the food we bought just didn’t last and we didn’t have money to get more.” In its validation cohort, an affirmative response to either question yielded a sensitivity of 97% and specificity of 83%, compared to the gold-standard, full 18-item HFSS.

If deemed food insecure by this screening process, providers will then order a “referral for food” in the EMR to connect the patient to Second Harvest Heartland for support. The patient must consent to the referral and state what specific contact information they are comfortable sharing with the partner organization. This referral provides the patient’s contact information to Second Harvest Heartland through an automated fax. The food bank staff will then assist the patient in enrolling in federal nutrition programs (see Table 1 for details), in addition to locating their neighborhood food shelves and meal programs, and arranging free produce distribution that they can access on a monthly basis.

**Population Health Research Capsule**

What do we already know about this issue?

The prevalence of hunger and the impact of food insecurity in the ED is a clinically important issue and likely underestimated. Food insecurity is prevalent in the U.S. and affected 12.7% of the population in 2016.

What was the research question?

The purpose of the study was to report the use of an integrated electronic medical record order for food resources and to describe our initial referral patterns.

What was the major finding of the study?

After focused education, ED referrals to the regional food bank increased from five in 2015 to 55 in 2016. Hospital-wide referrals increased from 1,003 in 2015 to 1,519 in 2016.

How does this improve population health? *The implementation of an integrated order in the electronic medical record for food resources is plausible and may increase ED use of food security resources.*

Though initially intended for clinic and inpatient use, starting in 2015 this order became available for use in the ED (Figure). To advertise the referral program, focused information sessions were added to the emergency medicine resident educational conferences in late 2015, and to the new resident orientation, starting in 2016. In addition to this, semi-annual updates are integrated into the resident conferences, and the details of the referral patterns are distributed to faculty. Laminated placards were distributed in the ED to encourage discussions with patients about food security. All ED personnel were encouraged to use the referral order, including ED faculty physicians, residents, physician assistants, nursing staff, social workers, ED registration, and financial support staff. Institutional financial counselors were unforeseen allies with the program, as their workflow typically incorporated several questions that touched on financial and food security issues.

### Data Analysis

All data analysis was descriptive, using counts and proportion as appropriate. We described the frequency of food resource referrals before and after the implementation of an ED EMR order for food resources.

## RESULTS

From January through December 2015 (preceding ED education on the EMR referral order), a total of 1,003 referrals were made to Second Harvest Heartland; only five were made from the ED. From January 2016 through December 2016 (after education regarding the EMR referral), there were 1,519 referrals hospital-wide, and 55 referrals were made from the ED. Table 2 outlines details of the frequency of EMR order use from all clinical sites. Of the 1,519 referrals, 1,129 (74%) were successfully contacted by Second Harvest Heartland, and 954 (63%) accepted and received assistance. Of the referred and successfully contacted households, 92% were connected with at least one new form of food assistance. This assistance included new information about geographically individualized food shelves, meal sites, and produce distribution. Of households eligible for the Supplemental Nutrition Assistance Program, 76% completed applications to the federal entitlement program. Additional details of the type of assistance patients received is detailed in Table 3.

## DISCUSSION

This study sought to determine whether ED referrals for food resources for patients with food insecurity would increase after the implementation of an EMR referral order, as well as to introduce provider education about this referral program. To our knowledge, we report the first experience with such an institutional EMR order for food resources in the ED. While previous studies have documented the prevalence and ramifications of food insecurity in the ED,^4^–^7^ the availability of a provider-driven order to improve this condition has not been previously documented.

Although food resources are available through a variety of federal and state programs, healthcare providers may be unaware of how to successfully connect patients with these programs. 10,11 Additionally, the details of the different programs, and understanding which programs apply to whom, can be unclear to patients and healthcare providers alike. Therefore, we believe that referring those patients in need to partners such as Second Harvest Heartland will likely be of greatest benefit to the patients, as these partners focus on one-on-one application assistance and navigation of programs, rather than simply handing out brochures or blank applications in the ED.

It is not surprising that using an EMR referral tool improved access to food services in our patient population; the benefit of EMR communication for connecting patients to numerous types of medical and social services has been well documented in the literature. 12–14 We did, however, identify certain issues with this referral process that are unique to food referrals and unique to the ED. For example, in contrast to the clinic setting where demographics and contact information is updated prior to patient evaluation, in the ED this information is frequently incomplete early in the patient’s visit. If the EMR order was placed without accurate contact information, the information provided to Second Harvest Heartland was also incomplete. In the early stages of the ED referral, this led to a disproportionate number of ED referrals lacking the necessary contact information and thus these patients could not be reached. After identifying this problem, the EMR order was changed, requiring the provider to enter an address, phone number, mobile number, or email address, ensuring proper communication to the food bank for follow-up. Ongoing, focused education was valuable in ensuring this aspect of the order was completed for successful referrals.

Another important consideration identified during the implementation of this process was realizing the knowledge gaps regarding food insecurity in our ED. Screening for food insecurity is not standardized at intake, nor is it part of the registration/rooming process. As such, in faculty and resident discussions surrounding use of the order, failure to consider food security as part of the ED assessment of patients was perceived to be a key limiting factor in making the food referral. Second Harvest Heartland began systematically visiting clinics and educating staff directly regarding the EMR order; while this helped increase referral volume in the clinics, the ED was targeted later in the rollout. We believe that this highlights the importance of provider education in the ED, as this patient population is at great risk for food insecurity and their needs may not be identified if they are not screened or if they do not use the clinic system. Even with education, concerted efforts and ongoing education are necessary.

## LIMITATIONS

This study was subject to certain limitations, as it was only intended to describe referral patterns before and after implementing an institutional EMR order for food resources. We do not have patient data, such as demographics, as the data we present was provided from the food bank and thus protected. We also cannot account for whether referral increases were due to secular trends, rather than the provider education and the EMR order, though this is unlikely given the magnitude of the increase seen. We also acknowledge that 55 referrals in 12 months is not a substantial number given the prevalence of food insecurity; 5,6 we do believe, however, that our findings are still notable, demonstrating that a 10-fold increase in referrals out of the ED was possible with just a simple provider-driven process change. Finally, we cannot speak to long-term outcomes and whether the referrals lead to meaningful use of food resources over time. We were able to report the frequency at which services were initiated from the referral, but not if the patients maintained those services successfully.

## CONCLUSIONS

This study suggests that using the EMR as a tool to refer patients to partner organizations for food resources is feasible, and may increase the frequency of referrals for food resources made from the ED. Such food-resource referrals are potentially an important initial step in addressing food insecurity in patients seen in the ED.

## Figures and Tables

**Figure:**
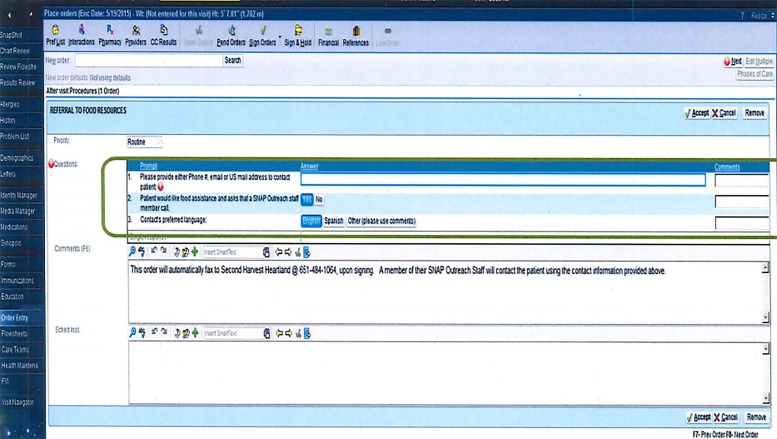
Emergency department “Order for Food Resources”.

**Table 1:** Most frequent electronic medical record orders for food resources

	Food resource referrals - 2016
Pediatrics clinic	438
Internal medicine clinic	140
Family medicine clinic	246
Emergency department	55 (25 Attending Physicians, 22 Resident Physicians, 8 Physician Assistants)
Psychiatry clinic	52

**Table 2:** List of food programs and whom they support

Name (acronym)	Description	Mothers & children	Seniors	Nutrition support	Foodbanks
Supplemental Nutrition Assistance Program (SNAP)	Formerly known as food stamps; now benefits are provided through an electronic card. SNAP can be used to purchase food at grocery stores.	X	X	X	
The Emergency Food Assistance Program (TEFAP)	The USDA buys food and distributes to individual states through food banks.	X	X	X	X
The Commodity Supplemental Food Program (CSFP)	Nutritious USDA foods are distributed to low-income seniors to supplement their diets.		X	X	X
The Child and Adult Care Food Program (CACFP)	Provides aid to child- and adult-care institutions with nutritious foods	X	X	X	X
The National School Lunch Program (NSLP)	Federally-assisted meal program providing nutritionally balanced, low-cost or free lunches to children in public and nonprofit private schools	X		X	
The School Breakfast Program (SBP)	Federally-assisted meal program providing nutritionally balanced, low cost or free lunches to children in public and nonprofit private schools. Some states have provided additional funding to provide universal, free breakfast for children.	X		X	
The Summer Food Service Program (SFSP)	A program that reimburses community organizations for hosting free meals for children under age 18 when they no longer have access to free and reduced-cost school lunch during the summer.	X		X	
Women, Infants, and Children (WIC)	Provides support for low-income, pregnant, breastfeeding and postpartum women and children up to age 5. This program is more nutrition-centered than SNAP as it includes check-ups, nutrition education, and supplemental foods.	X		X	

**Table 3:** Types of assistance received as a result of the EMR food referral order

Assistance type*	Number of eligible referrals	% Eligible referrals
Community based referrals		
Food shelf	829	88%
Fare for all	825	88%
SFSP / community meals	742	79%
NAPS	96	10%
WIC	45	4%
SNAP screenings		
Already on SNAP	508	54%
SNAP application assistance		% of 446 patients not previously enrolled
		
Completed applications	338	76%
Ineligible	99	22%
Not interested in applying	9	2%

*EMR*, electronic medical record; *SFSP*, Summer Food Service Program; *NAPS*, Nutritional Assistance Program for Seniors; *WIC*, Women, Infants and Children; *SNAP*, Supplemental Nutritional Assistance Program.

* May be eligible for one or more.
